# Correction: Effect of non-covalent self-dimerization on the spectroscopic and electrochemical properties of mixed Cu(i) complexes

**DOI:** 10.1039/d3ra90033a

**Published:** 2023-04-11

**Authors:** Joaquín Cáceres-Vásquez, Danilo H. Jara, Juan Costamagna, Fabián Martínez-Gómez, Carlos P. Silva, Luis Lemus, Eleonora Freire, Ricardo Baggio, Cristian Vera, Juan Guerrero

**Affiliations:** a Laboratorio de Compuestos de Coordinación y Química Supramolecular, Facultad de Química y Biología, Universidad de Santiago de Chile Av. Libertador Bernardo O’Higgins 3363, Estación Central, Casilla 40, Correo 33 Santiago Chile juan.guerrero@usach.cl cristian.veraoy@usach.cl; b Facultad de Ingenieria y Ciencias, Universidad Adolfo Ibáñez Av. Padre Hurtado 750 Viña del Mar Chile; c Gerencia de Investigación y Aplicaciones, Centro Atómico Constituyentes, Comisión Nacional de Energía Atómica Avenida Gral. Paz 1499, 1650, San Martín Buenos Aires Argentina baggio@tandar.cnea.gov.ar; d Escuela de Ciencia y Tecnología, Universidad Nacional de San Martín, Argentina and Gerencia de Investigación y Aplicaciones, Centro Atómico Constituyentes, Comisión Nacional de Energía Atómica Buenos Aires Argentina; e Member of CONICET Argentina; f Facultad de Química y Biología, Universidad de Santiago de Chile Av. Libertador Bernardo O’Higgins 3363, Estación Central, Casilla 40, Correo 33 Santiago Chile

## Abstract

Correction for ‘Effect of non-covalent self-dimerization on the spectroscopic and electrochemical properties of mixed Cu(i) complexes’ by Joaquín Cáceres-Vásquez *et al.*, *RSC Adv.*, 2023, **13**, 825–838, https://doi.org/10.1039/D2RA05341A.

The authors regret that the funding information was incorrectly shown in the Acknowledgements section of the original manuscript. The corrected funding acknowledgement is as shown below.

The authors acknowledge the support of DICYT-USACH, FONDECYT 1191902, and FONDEQUIP-EQM 150106. C. V. acknowledges support from postdoctoral grant FONDECYT 3170661, J. C.-V. acknowledges a CONICYT Doctoral Fellowship 21141067.

The Royal Society of Chemistry apologises for these errors and any consequent inconvenience to authors and readers.

## Supplementary Material

